# Efficacy of Ultrasound-Guided Serratus Anterior Plane Block for Postoperative Analgesia in Patients Undergoing Breast Surgery: A Systematic Review and Meta-Analysis of Randomised Controlled Trials

**DOI:** 10.1155/2021/7849623

**Published:** 2021-10-25

**Authors:** Nian-Qiang Hu, Qi-Qi He, Lu Qian, Ji-Hong Zhu

**Affiliations:** ^1^Department of Anesthesiology, Sir Run Run Shaw Hospital, School of Medicine, Zhejiang University, Hangzhou, China; ^2^Department of Nursing, Sir Run Run Shaw Hospital, School of Medicine, Zhejiang University, Hangzhou, China

## Abstract

**Objective:**

Serratus anterior plane block (SAPB) provides effective thoracic analgesia. This systematic review and meta-analysis was conducted to assess the safety and efficacy of SAPB for postoperative analgesia after breast surgery.

**Methods:**

A systematic literature search was performed using Embase, PubMed, Web of Science, and the Cochrane Library for eligible randomised controlled trials. The primary outcomes involved the administration of intraoperative and postoperative opioids. The Grading of Recommendations, Assessment, Development and Evaluation (GRADE) approach was used for rating the quality of evidence for making recommendations.

**Results:**

Overall, 13 studies comprising 826 patients met the inclusion criteria (412 in the SAPB group and 414 in the control group). Patients treated with SAPB exhibited a significantly lower postoperative opioid consumption (mean difference, −38.51 mg of oral morphine equivalent; 95% confidence interval (CI), −60.97 to −16.05; *P* < 0.01; *I*^*2*^ = 100%), whereas no difference was observed in the intraoperative opioid consumption (mean difference, −9.85 mg of oral morphine equivalent; 95% CI, −19.52 to −0.18; *P*=0.05; *I*^*2*^ = 94%). In addition, SAPB significantly decreased the occurrence of postoperative nausea and vomiting (risk ratio, 0.32; 95% CI, 0.19–0.55; *P* < 0.05;*I*^*2*^ = 38%) and reduced pain scores during the postoperative period (1 h: standardised mean difference (SMD), −1.23; 95% CI, −2.00 to −0.45; *I*^*2*^ = 92%; 2 h: SMD, −0.71; 95% CI, −1.00 to −0.41; *I*^*2*^ = 48%; 4 h: SMD, −1.52; 95% CI, −2.77 to −0.27; *I*^*2*^ = 95%; 6 h: SMD, −0.80; 95% CI, −1.51 to −0.08; *I*^*2*^ = 81%; 8 h: SMD, −1.12; 95% CI, −1.98 to −0.27; *I*^*2*^ = 92%; 12 h: SMD, −0.78; 95% CI, −1.21 to −0.35; *I*^*2*^ = 83%; and 24 h: SMD, −0.71; 95% CI, −1.20 to −0.23; *I*^*2*^ = 87%; *P* < 0.05 for all).

**Conclusion:**

SAPB was safe and effective after breast surgery to relieve postsurgical pain. However, additional well-developed trials are required to validate these findings.

## 1. Introduction

According to global public health data, the most common type of cancer affecting women is breast cancer [1]. Surgical removal of the tumour is the primary treatment for breast cancer. However, postoperative pain continues to pose a problem in patients with breast cancer. Approximately 50% of patients who undergo breast surgery experience some degree of postoperative pain [2, 3]. Severe pain hampers postoperative recovery [4] and prolongs the hospital stay [5]. In addition, the risk of progression of acute pain after breast surgery to chronic pain continues to remain [2]. Therefore, various techniques for analgesia such as intercostal block [6], erector spinae plane block [7], and paravertebral block [8] are reported to ease severe postsurgical pain.

Serratus anterior plane block (SAPB) is a recently described interfascial plane block technique to relieve thoracic pain by injecting a local anaesthetic into the plane between the latissimus dorsi muscle and serratus anterior muscle [9]. SAPB provides effective postoperative analgesia by blocking the lateral cutaneous branches of the thoracic intercostal nerves. Previous studies have demonstrated that SAPB can be used as a locoregional analgesic technique to reduce pain after breast surgery [10, 11]. However, the effectiveness of this method for inducing analgesia remains controversial. A recent randomised controlled trial (RCT) indicated that a deep SAPB did not have any beneficial effects on postoperative analgesic outcomes such as pain scores and opioid consumption [12]. However, no systematic and convincing proof related to this has been reported.

Therefore, we performed this systematic review and meta-analysis of RCTs to explore the safety and effectiveness of the SAPB technique in breast surgery.

## 2. Methods

The present systematic review and meta-analysis was performed according to the guidelines of Preferred Reporting Items for Systematic Reviews and Meta-Analyses (PRISMA).

### 2.1. Systematic Literature Search

A systematic literature search was conducted using online databases, including Embase, PubMed, the Cochrane Library, and Web of Science, from the date of the establishment of the database to 31 March 2021 without any language restriction, and relevant RCTs were identified. The PubMed search criteria were as follows: (1) “serratus anterior block” (All Fields), “serratus anterior plane block” (All Fields), or “SAP block” (All Fields), (2) “sap block” (All Fields), “SAPB” (All Fields), and (3) “breast surgery” (All Fields), “breast cancer” (All Fields), “breast” (All Fields), or “breasts” (All Fields). Search strategies for other databases are shown in Supplementary Materials. Furthermore, we manually searched for the references in the relevant literature.

### 2.2. Selection Criteria and Data Extraction

Studies meeting the following inclusion criteria were included in this analysis: (1) participants: studies involving patients undergoing breast surgery; (2) intervention: studies clearly describing SAPB as an auxiliary technique for analgesia regardless of the timing of placement of the regional block before or after general anesthesia; (3) comparison: studies with no intervention, sham block, or incision infiltration; (4) outcome: studies reporting opioid consumption or postoperative pain score; and (5) study design: RCTs. The following articles were excluded from this study: (1) review or case reports; (2) trials on animals or research involving cadaver dissection; (3) conference abstracts; and (4) duplicate publications.

EndNote X9 was used to pick out authentic trials from duplicate ones. Two different authors checked the authenticity of the article titles and abstracts and carefully assessed the full texts to ensure that the articles met the eligibility criteria for this study. The data were collected from authentic publications and were independently cross-checked by the two authors. The data collected included the first author's name, sample size, age, surgery type, SAPB, general anesthesia techniques, comparison, postoperative opioids analgesia, and pain measurements. For trials with incomplete data, corresponding authors were contacted via e-mail to obtain the complete information.

### 2.3. Quality and Risk Assessment

The risk of bias in the publications included in this study was assessed using Cochrane Review Manager (Version 5.3; the Nordic Cochrane Centre, the Cochrane Collaboration, Copenhagen, Denmark, 2014). We used the following methods to assess bias: random sequence generation, allocation concealment, incomplete outcome data, double blinding, blinding of outcome assessment, and selective reporting. Each study was individually analysed by two reviewers and was classified into three groups: low risk, unclear risk, and high risk.

The quality of evidence was examined using the Grading of Recommendations Assessment, Development and Evaluation (GRADE) system to obtain results as per the following criteria: study design, inconsistency rating in results, risk of bias, and rating of indirectness of evidence. The quality of evidence was classified into 4 groups as high, moderate, low, and very low.

### 2.4. Primary and Secondary Outcomes

Intraoperative and postoperative 24 h opioid consumptions were the primary outcomes. The dose of different types of opioids consumed was converted to an equivalent dose of oral morphine according to GlobalRPh (http://www.globalrph.com/narcotic). Secondary outcomes included the occurrence of adverse events and scores of postsurgical pain at different time points. Results from a previous study showed a correlation between the scores obtained on the visual analogue scale (VAS) and the numeric rating score (NRS) [13]; therefore, we analysed the pain scores obtained using these two scales. For studies that measured pain scores at different states, active pain scores were included in this meta-analysis.

### 2.5. Statistical Analysis

The meta-analysis was performed using Review Manager (version 5.3; the Nordic Cochrane Centre, the Cochrane Collaboration, Copenhagen, Denmark, 2014) and Stata V.12.0 (StataCorp LP, USA). A pooled risk ratio (RR) and 95% confidence intervals (CIs) were calculated for dichotomous outcomes. *P* < 0.05 was considered significant. Mean difference (MD) and 95% confidence interval (CI) were measured using the same units for continuous data, whereas standardised mean difference (SMD) was used for different units. The data reported as medians (ranges) were converted to mean and standard deviation similar to those performed in previous studies [14, 15]. We assessed heterogeneity among trials using the statistic. High heterogeneity may be observed because of methodological and clinical factors; therefore, despite the low value, the random effects model was implemented in this meta-analysis. Sensitivity analysis was performed to assess the stability of the primary outcome.

## 3. Results

### 3.1. Search Results

**Figure 1:**
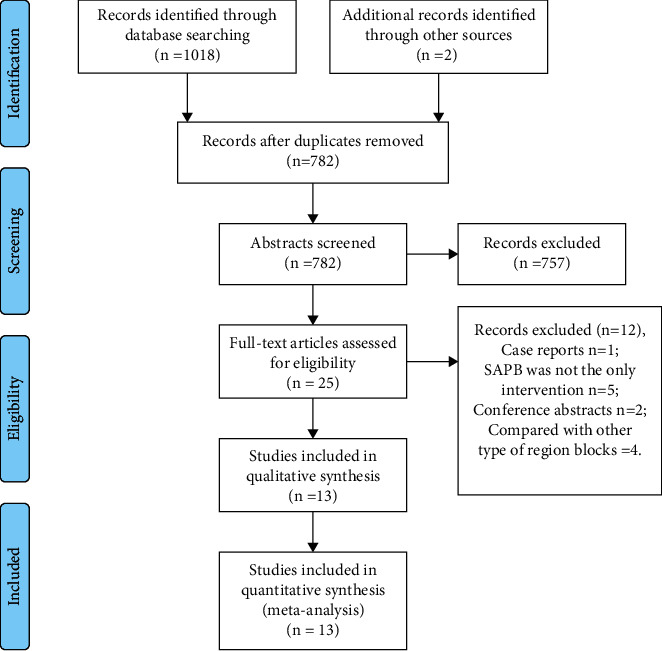
Flow chart of studies retrieval.

On the basis of our search strategy, we identified 1020 relevant trials. Among them, 238 trials were duplicate publications and 757 were excluded because after screening their abstracts, they were found to be irrelevant for this meta-analysis. Moreover, the remaining 25 full-text publications were carefully evaluated to check their eligibility. Furthermore, 12 trials were eliminated owing to the following reasons: they were case reports (*n* = 1) [16]; SAPB was not the only intervention (*n* = 5) [17–21]; they were conference abstracts (*n* = 2) [10, 22]; and SAPB was compared with other types of nerve blocks (*n* = 4) [11, 23–25]. Eventually, we included 13 eligible and authentic studies [12, 26–37] in this meta-analysis. The screening process for the literature is shown in Figure 1.

### 3.2. Study Characteristics

We analysed 13 RCTs consisting of 826 subjects who underwent breast surgery. The years of publication of these studies ranged from 2017 to 2021, and the sample size ranged from 40 to 116. Overall, 7 trials, 5 trials, and 1 trial used bupivacaine [26, 27, 30, 32, 34, 36], ropivacaine [12, 29, 33, 35, 37], and levobupivacaine [28] as a local anaesthetic, respectively. The volume of the local anaesthetic was 20–40 mL, and its concentration was 0.25%–0.5%. Overall, 12 trials evaluated pain scores using VAS, whereas 1 trial used NRS [35]. The details about the trials included in this meta-analysis are shown in Table 1.

### 3.3. Assessment of Bias

**Figure 2:**
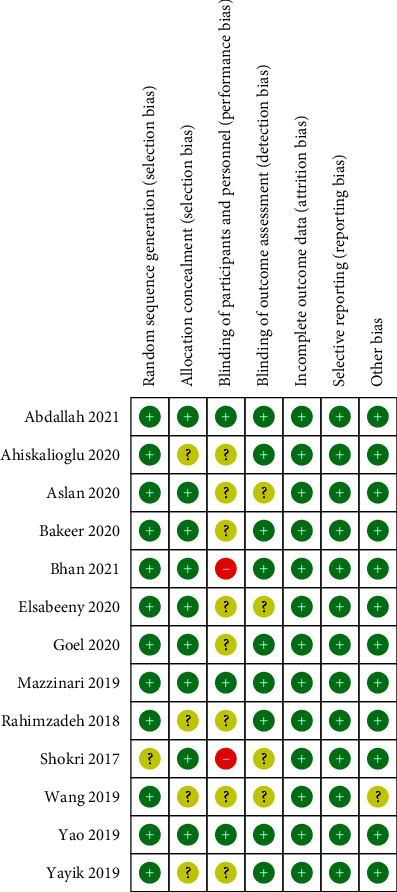
Risk bias of included studies.

A total of 10 trials used the random sequence generation method [12, 27–37], 9 trials explained the allocation concealment [12, 26, 28, 30, 33, 35, 36], 3 trials explicitly described the method of double blinding [12, 28, 36], and 9 trials described blinded assessors and evaluated attrition bias [12, 27, 28, 31, 33–37]. There was no selective reporting. The sample size of one trial [29] was not measured, and the other bias was classified into an unclear group. There were no reports of other biases, such as trial registration. The risk of bias is explained briefly in Figure 2.

### 3.4. Primary Outcomes

**Figure 3:**
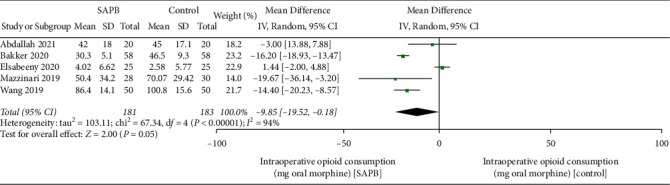
The forest plot of pooled analysis showing intraoperative opioid consumption.

**Figure 4:**
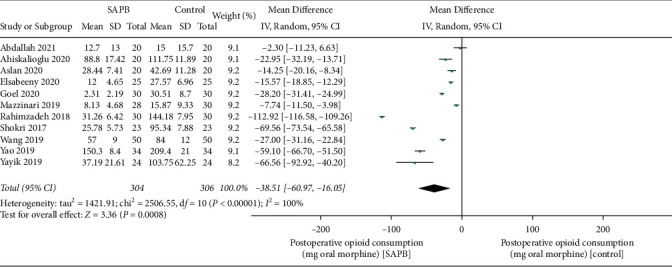
The forest plot of pooled analysis showing postoperative opioid consumption.

Intraoperative consumption of opioids was reported in 5 trials. The outcome showed a negligible difference between the two groups (MD, −9.85 mg of oral morphine equivalent; 95% CI, −19.52 to −0.18; *P*=0.05; *I*^*2*^ = 94%; Figure 3). Opioid consumption during the first postoperative 24 h was assessed in 11 trials. The forest plot data indicated that SAPB significantly decreased postoperative opioid consumption (MD, −38.51 mg of oral morphine equivalent; 95% CI, −60.97 to −16.05; *P* < 0.01;  = 100%; Figure 4).

### 3.5. Secondary Outcomes

**Figure 5:**
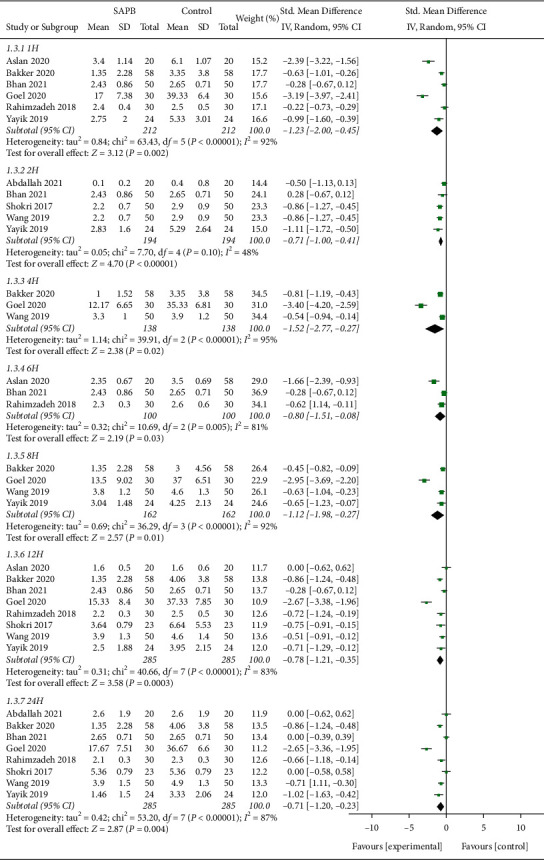
The forest plot of pooled analysis showing postoperative pain scores (H, hour).

Pain scores were evaluated at different time points during the first postoperative 24 h. A forest plot showed that SAPB could significantly relieve postoperative pain (1 h: SMD, −1.23; 95% CI, −2.00 to −0.45; *P* < 0.05;  = 92%; 2 h: SMD, −0.71; 95% CI, −1.00 to −0.41; *P* < 0.05;  = 48%; 4 h: SMD, −1.52; 95% CI, −2.77 to −0.27; *P* < 0.05; *I*^*2*^ = 95%; 6 h: SMD, −0.80; 95% CI, −1.51 to −0.08; *P* < 0.05;  = 81%; 8 h: SMD, −1.12; 95% CI, −1.98 to −0.27; *P* < 0.05;  = 92%; 12 h: SMD, −0.78; 95% CI, −1.21 to −0.35; *P* < 0.05;  = 83%; and 24 h: SMD, −0.71; 95% CI, −1.20 to −0.23; *P* < 0.05;  = 87%; Figure 5).

**Figure 6:**
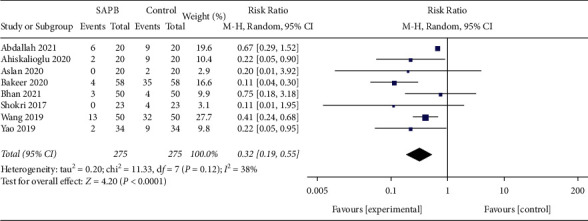
The forest plot of pooled analysis showing the incidence of postoperative nausea and vomiting.

Sickness and vomiting were reported after surgery in 8 trials (postoperative nausea and vomiting, PONV). A forest plot showed a significantly low occurrence of PONV in the SAPB group (RR, 0.32; 95% CI, 0.19–0.55; *P* < 0.05;  = 38%; Figure 6). Procedure-related complications were not reported in the trials included in this analysis.

### 3.6. Publication Bias

We did not evaluate the publication bias because only a few studies were included in this analysis [38].

### 3.7. Sensitivity Analysis

**Figure 7:**
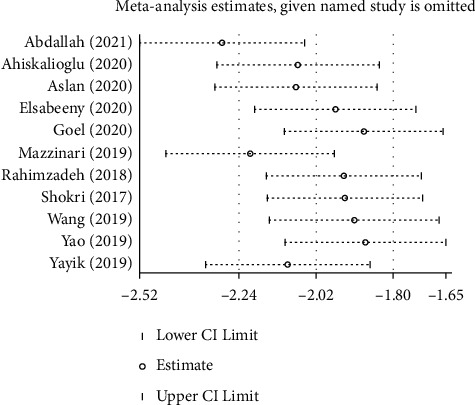
Sensitivity analysis for postoperative opioid consumption.

Sensitivity analysis was performed for postoperative opioid consumption. The estimate of effect did not change, indicating the robustness of the formulated result (Figure 7).

### 3.8. GRADE Assessment

All studies included were RCTs. Most studies showed a relatively high . The “inconsistency” was classified as serious. Some trials reported median pain scores and opioid consumption. The “indirectness” was graded as serious. The GRADE levels were low and moderate for the outcomes. The total outcomes of the GRADE assessment are concisely shown in Table 2.

## 4. Discussion

This systematic review and meta-analysis revealed that ultrasound-guided SAPB decreased opioid consumption (low quality) to a significant level and relieved pain after breast surgery (low quality). In addition, SAPB decreased the occurrence of PONV (moderate quality). Procedure-related complications were not seen in the studies included in the analysis.

Patients undergoing breast cancer surgery experience varying degrees of acute and chronic pain with an incidence of up to 60% [39]. Various analgesic techniques have been used in breast surgeries, including paravertebral block [8], intercostal block [6], and pectoral nerve block [40]. Paravertebral block and intercostal nerve block are associated with a risk of development of pneumothorax (0.3%–11.4%); therefore, these methods are not the preferred choice of anaesthesiologists [41, 42]. Effective control of pain after breast surgery is very important not only to more effectively manage acute pain but also to improve postoperative recovery [43].

The development of ultrasound-guided regional block and the introduction of new regional analgesia techniques have increased the safety and effectiveness of perioperative analgesia for thoracic surgery. Ultrasound-guided SAPB is a recently described regional block technique. It involves the injection of a local anaesthetic into the region between the serratus anterior and intercostal muscles [9]. The thoracic region from T2 to T9 is blocked using this technique. A previous meta-analysis [44] reported that SAPB might provide effective analgesia after breast surgery; however, the control group in that meta-analysis included patients who received a paravertebral block.

The results of our meta-analysis revealed that SAPB remarkably decreased the levels of postoperative opioid consumption and decreased pain compared with that in the control group, which indicated that SAPB could provide effective analgesia after breast surgery. However, a recent RCT [12] showed that SAPB did not improve analgesic outcomes in patients who underwent ambulatory breast cancer surgery. This could be because “breast surgery,” as the type of surgery being performed, is too generic a term and involves all degrees of trespass; therefore, comparison of the results obtained in this study with our results may be difficult. Moreover, deep SAPB alone was performed in a previous study, and Mayes et al. [45] reported that injecting methylene blue deep into the serratus anterior muscle did not produce a consistent spread area. The results of our meta-analysis revealed no difference in intraoperative opioid consumption between the two groups. This finding may be because the extent of SAPB was not sufficient to control intraoperative pain. Results similar to those of our study were reported by Alessandro et al. in patients undergoing thoracoscopic surgery [46].

In addition, we observed that patients treated with SAPB had a significantly lower incidence of PONV. This may be a result of less opioid usage during the postoperative period. The results of a recent RCT with a large sample indicated that the incidence of PONV was 44.3% after general anesthesia [47]. Effective prevention of PONV is important in the implementation of enhanced recovery after surgery. Reduction of PONV is useful in expediting discharge for outpatients [48].

Procedure-related complications were not seen in the studies included in this analysis. SAPB is a relatively safe technique of regional block, and potential block-related complications such as bleeding and infection at the puncture site have not been reported. To date, only a case report by Desai described the development of pneumothorax following SABP in a patient undergoing wire-guided wide local excision of a lump in the right breast [49]. However, as a novel regional block technique, high-quality trials are required to ensure the safety of SAPB.

Our meta-analysis had some limitations. The analysis was performed with a limited number of participants. Therefore, studies with a larger sample size should be performed in the future. Furthermore, a high level of clinical heterogeneity may be present because of the various general and local anesthetics administered to the patients. We were unable to compare the advantages and disadvantages of SAPB with other techniques of regional anesthesia because of an insufficient number of RCTs pertaining to this topic.

## 5. Conclusion

SAPB is safe and effective in inducing postoperative analgesia after breast surgery. However, well-designed trials are required to validate these findings.

## Figures and Tables

**Table 1:** The characteristics of included studies.

Study	Sample size	Age (years)	Type of surgery	General anesthesia	SAPB technique	Control group	Postoperative opioid analgesia	Pain measurement
Abdallah 2021	S: 20	18–80	Unilateral partial or simple mastectomy	Induction: fentanyl 1–3 *μ*g/kg, propofol 2–4 mg/kg, and rocuronium 0.6 mg/kg	Position: lateral decubitus; local anesthetics: 20 ml of 0.5% ropivacaine	Sham block:1 ml sterile saline subcutaneously	Fentanyl intravenous; hydromorphone intravenous; and oxycodone oral intake	VAS
	C: 20			Maintenance: desflurane 2–6% in a 50 : 50 mixture of oxygen and air	Timing: before the general anesthesia			
Ahiskalioglu 2020	S: 20	18–60	Breast reduction surgery	Induction: fentanyl 1–2 *μ*g/kg, propofol 2 mg/kg, and rocuronium 0.6 mg/kg	Position: lateral decubitus; local anesthetics: 30 ml of 0.25% bupivacaine	Sham block: 2 ml saline was injected subcutaneously	Fentanyl PCA	VAS
	C: 20			Maintenance: sevoflurane1–2% in a 50 : 50 mixture of oxygen and N_2_O	Timing: before the general anesthesia			
Aslan 2020	S: 20	18–70	Modified radical mastectomy	Induction: fentanyl 1 *μ*g/kg, propofol 2–3 mg/kg, and rocuronium 0.6 mg/kg	Position: supine position; local anesthetics: 40 ml of 0.25% bupivacaine	No block	Morphine PCA	VAS
	C: 20			Maintenance: 48% nitrogen oxide, 2% sevoflurane, and 50% oxygen	Timing: after the general anesthesia			
Bakeer 2020	S: 58	18–60	Unilateral modified radical mastectomy	Induction: fentanyl 1 *μ*g/kg, propofol 2 mg/kg, and cisatracurium 0.15 mg/kg	Position: lateral position; local anesthetics: 30 ml of 0.25% bupivacaine	No block	Morphine intravenous	VAS
	C: 58			Maintenance: 2% sevoflurane in 50% mixture of oxygen and air	Timing: before the general anesthesia			
Bhan 2021	S: 50	18–65	Modified radical mastectomy	Induction: fentanyl 2 *μ*g/kg, propofol 1–2 mg/kg, and vecuronium 0.1 mg/kg	Position: supine position; local anesthetics: 0.4 mL kg-1 of 0.375% ropivacaine (maximum volume of 30 mL)	No block	Other analgesia drugs	NRS
	C: 50			Maintenance: 1 minimum alveolar concentration desflurane in oxygen and air	Timing: before the general anesthesia			
Elsabeeny 2020	S: 25	18–65	Modified radical mastectomy	Induction: fentanyl 2 *μ*g/kg, propofol 2 mg/kg, and rocuronium 0.6 mg/kg	Position: lateral position; local anesthetics: 25 ml of 0.25% bupivacaine	No block; morphine sulphate 0.1 mg/kg	Morphine intravenous	VAS
	C: 25			Maintenance: sevoflurane and rocuronium	Timing: after the general anesthesia			
Goel 2020	S: 30	20–80	Modified radical mastectomy	Induction: propofol 2 mg/kg, morphine 0.1 mg/kg, and vecuronium 0.1 mg/kg	Position: NR local anesthetics; 20 ml of 0.2% ropivacaine	No block	Morphine PCA	VAS
	C: 30			Maintenance: NR	Timing: after the general anesthesia			
Mazzinari 2019	S: 28	≥18	Oncologic breast surgery	Induction: midazolam 0.01–0.03 mg/kg, fentanyl 1 *μ*g/kg, propofol 2 mg/kg, and rocuronium bromide 0.6 mg/kg	Position: NR; local anesthetics: 30 ml of 0.25% levobupivacaine	No block	Morphine PCA	VAS
	C: 30			Maintenance: propofol	Timing: after the general anesthesia			
Rahimzadeh 2018	S: 30	20–60	Modified radical mastectomy	Induction: NR	Position: lateral decubitus position; local anesthetics: 0.3 ml/kg of 0.2% bupivacaine	No block	Fentanyl PCA	VAS
	C: 30			Maintenance: NR	Timing: after the general anesthesia			
Shokri 2017	S: 23	40–56	Breast surgeries	Induction: fentanyl 2 *μ*g/kg, thiopentone sodium 3–5 mg/kg, and atracurium 0.5 mg/kg	Position: supine position; local anesthetics: 0.4 ml/kg of 0.25% bupivacaine plus 20 *μ*g fentanyl	Incision infiltration: 0.4 ml/kg of 0.25% bupivacaine and 20 *μ*g fentanyl	Pethidine intravenous	VAS
	C: 23			Maintenance: isoflurane and atracurium	Timing: before the general anesthesia			
Wang 2019	S: 50	NR	Radical mastectomy	Induction: midazolam 0.02 mg/kg, sufentanil 0.4 *μ*g/kg, propofol 2 mg/kg, and cisatracurium 0.2 mg/kg	Position: lateral position; local anesthetics: 20 ml of 0.375% ropivacaine	No block	Sufentanil PCA	VAS
	C: 50			Maintenance: Propofol and remifentanil	Timing: before the general anesthesia			
Yao 2019	S: 34	18–60	Unilateral breast cancer surgery	Induction: sufentanil 0.5 *μ*g/kg, propofol 2 mg/kg, and cisatracurium 0.15 mg/kg	Position: lateral position	Sham block: physiological saline	Sufentanil PCA	VAS
	C: 34			Maintenance: sevoflurane	Local anesthetics: 25 ml of 0.5% ropivacaine; timing: before the general anesthesia			
Yayik 2019	S: 24	18–65	Modified radical mastectomy	Induction: fentanyl 1–2 *μ*g/kg, propofol 2 mg/kg, and rocuronium 0.6 mg/kg	Position: lateral position; local anesthetics: 20 ml of 0.25% bupivacaine	Sham block: 2 ml saline was injected subcutaneously	Fentanyl PCA	VAS
	C: 24			Maintenance: sevoflurane1–2% in a 50 : 50 mixture of oxygen and N2O	Timing: before the general anesthesia			

SAPB, serratus anterior plane block; S, serratus anterior plane block group; C, control group; VAS, visual analogue scale; NRS, numeric rating score; PCA, patient-controlled analgesia devices; NR, not reported.

**Table 2:** The summary of the GRADE evaluation.

Outcome	MD/SMD/RR (95% CI)	Quality of evidence	Reasons
Intraoperative opioid consumption	−9.85 (−19.52, −0.18)	⨁⨁◯◯ LOW	Indirectness was “serious”;;inconsistency was “serious”
Postoperative opioid consumption	−38.51 (−60.97, −16.05)	⨁⨁◯◯ LOW	Indirectness was “serious”;inconsistency was “serious”
Pain score at 1 H postoperatively	−1.23 (−2.00, −0.45)	⨁⨁◯◯ LOW	Indirectness was “serious”; inconsistency was “serious”
Pain score at 2 H postoperatively	−0.71 (−1.00, −0.41)	⨁⨁◯◯ LOW	Indirectness was “serious”; inconsistency was “serious”
Pain score at 4 H postoperatively	−1.52 (−2.77, −0.27)	⨁⨁◯◯ LOW	Indirectness was “serious”; inconsistency was “serious”
Pain score at 6 H postoperatively	−0.80 (−1.51, −0.08)	⨁⨁◯◯ LOW	Indirectness was “serious”; inconsistency was “serious”
Pain score at 8 H postoperatively	−1.12 (−1.98, −0.27)	⨁⨁◯◯ LOW	Indirectness was “serious”; inconsistency was “serious”
Pain score at 12 H postoperatively	−0.78 (−1.21, −0.35)	⨁⨁◯◯ LOW	Indirectness was “serious”; inconsistency was “serious”
Pain score at 24 H postoperatively	−0.71 (−1.20, −0.23)	⨁⨁◯◯ LOW	Indirectness was “serious”; inconsistency was “serious”
Incidence of PONV	0.32 (0.19, 0.55)	⨁⨁⨁◯ MODERATE	Inconsistency was “serious”

SMD, standardised mean difference; RR, risk ratio; H, hour; PONV, postoperative nausea and vomiting.

## Data Availability

All data generated or analysed during this study are included within this published article and its supplementary information files.

## Supplementary Materials

Supplementary Materials Search strategies for other databases. .
