# Supplementary material for: Efficacy of Ultrasound-Guided Serratus Anterior Plane Block for Postoperative Analgesia in Patients Undergoing Breast Surgery: A Systematic Review and Meta-Analysis of Randomised Controlled Trials
Source: Pain Res Manag. 2021 Oct 25;2021:7849623. doi: 10.1155/2021/7849623 (PMC8560299; doi:10.1155/2021/7849623)
Supplement: Supplementary Materials — Search strategies for other databases. . [file 7849623.f1.zip › 7849623.f1/Supplemental Material.docx]

Search strategy for Web of Science

1# TS=(serratus anterior block) OR TS=(serratus anterior plane block) OR TS=(sap block) OR TS= (SAP block) OR TS= (SAPB)

2# TS=(breast surgery OR breast cancer OR breast OR breasts).

1# AND 2#

Search strategy for the Cochrane Library

(“serratus anterior block” OR “serratus anterior plane block” OR “sap block”) AND (“breast surgery” OR “breast cancer” OR “breast” OR “breasts”).

Search strategy for EMBASE

#1 (serratus AND anterior AND block) OR (sap AND block) OR sapb OR (serratus AND anterior AND plane AND block) OR SAPB OR (SAP AND block)

#2 (breast AND surgery) OR (breast AND cancer) OR breast OR breasts

#1 AND #2
